# Isolation and identification of Tete virus group (*Peribunyaviridae*: *Orthobunyavirus*) from *Culicoides* biting midges collected in Lichuan County, China

**DOI:** 10.3389/fcimb.2023.1193184

**Published:** 2023-10-31

**Authors:** Qikai Yin, Rui Cheng, Xiuyan Xu, Ziqian Xu, Jing Wang, Shihong Fu, Hongbin Xu, Shaozai Zhang, Ying He, Fan Li, Songtao Xu, Xiaoqing Lu, Huanyu Wang, Bin Wang, Guodong Liang

**Affiliations:** ^1^ State Key Laboratory of Infectious Disease Prevention and Control, National Institute for Viral Disease Control and Prevention, Chinese Center for Disease Control and Prevention, Beijing, China; ^2^ School of Public Health, Qingdao University, Qingdao, China; ^3^ Luoyang City Center for Disease Control and Prevention, Luoyang, China; ^4^ Jiangxi Province Center for Disease Control and Prevention, Nanchang, China; ^5^ School of Basic Medicine, Qingdao University, Qingdao, China

**Keywords:** lichuan virus (LICV), Orthobunyavirus, Tete virus, Culicoides biting midges, evolution

## Abstract

In July 2018, a virus (JXLC1806-2) was isolated from *Culicoides* biting midges collected in Lichuan County, Jiangxi Province, China. The virus isolate showed significant cytopathic effects within 48 hours after inoculation with mammalian cells (BHK-21). JXLC1806-2 virus could form plaques in BHK-21 cells, and the virus titer was 1×10^5.6^ pfu/mL. After inoculation with the virus, suckling mice developed disease and died. The nucleotide and amino sequence analysis showed that the JXLC1806-2 virus genome was composed of S, M and L segments. Phylogenetic analysis showed that the S, M and L genes of JXLC1806-2 virus belonged to the Tete serogroup, *Orthobunyavirus*, but formed an independent evolutionary branch from the other members of the Tete serogroup. The results showed that the JXLC1806-2 virus, which was named as Lichuan virus, is a new member of Tete serogroup, and this is the first time that a Tete serogroup virus has been isolated in China.

## 1 Introduction

The virus classification report released by the International Committee on Taxonomy of Viruses (ICTV) in 2017 classified all viruses into 9 orders, 131 families, 46 subfamilies, 803 genera, and 4853 species (Lefkowitz et al., 2017). *Orthobunyavirus* is a viral genus of the *Peribunyaviridae* family in the *Bunyavirales* order. This family of viruses contains four viral genera, namely *Hebevirus, Orthobunyavirus, Pacuvirus* and *Shangavirus (* Abudurexiti et al., 2019). The representative virus of the *Orthobunyavirus* genus is the Bunyamwera virus (Shchetinin et al., 2015; Lefkowitz et al., 2017). Orthobunyaviruses are enveloped negative strand RNA viruses and contain three segments called large protein (L), medium protein (M), and small protein (S). Orthobunyaviruses like Akabane, Aino and Schmallenberg viruses can cause asymptomatic, or mild infection or congenital malformations in animals such as sheep, cattle, and horses. Virus infection in humans such as California encephalitis virus, La Crosse virus, Ngari virus, Oropouche virus and Nyando virus can cause clinical symptoms such as fever, hemorrhage, and encephalitis, and presents a substantial public health burden (Shchetinin et al., 2015; Lefkowitz et al., 2017). The transmission vectors of orthobunyaviruses include mosquitoes, ticks, and midges. As viruses carried by blood-feeding arthropods are disseminated and infectious to humans and various other animals, the isolation and identification of viruses from blood-feeding arthropods vectors represent not only an important aspect of virological research but is also a public health concern.

There are many types of orthobunyaviruses, and based on the serological reactions of the viruses, they can be divided into several serogroups (Shchetinin et al., 2015; Lefkowitz et al., 2017). The Tete serogroup belongs to the *Orthobunyavirus* genus, which includes the Tete virus (SAAn3518 virus strain), which was isolated from newborn mice inoculated with a bird (*Ploceus cucullatus*, spotted-back weaver-bird) tissue sample collected in South Africa on February 5, 1959. This was the first virus that was isolated of the Tete serogroup viruses (Taylor, 1967; Shchetinin et al., 2015). The Tete serogroup Bahig (EgB 90) (Balducci et al., 1973; Converse et al., 1974) and Matruh viruses (An 1047-61) (Balducci et al., 1973) were also isolated from bird (*Oriolus oriolus*) and ticks samples collected in Egypt and Cyprus in 1966. The I612045 virus was isolated in India in 1961 (NCBI, 2012), the Batama virus (AnB 1292a) was isolated from birds (*Euplectes afer*) collected in the Central African Republic in 1970 (Karabatsos, 1978), and the Weldona virus (76V-21935 and 77V 5691) was isolated from midges collected in Colorado, USA in 1990 (Calisher et al., 1990). China has isolated multiple strains of orthobunyaviruses from mosquito specimens collected from nature, including the Simbu serogroup Cat Que, Manzanilla, and Akabane viruses, as well as the California serogroup Tahyna virus (Lu et al., 2009; Feng et al., 2015; Zhang et al., 2015; Feng et al., 2016), but there have been no reports of the isolation of orthobunyaviruses from blood-feeding arthropods other than mosquitoes since the 1980s (Liang et al., 2017). In this study, the JXLC1806-2 virus was isolated in the summer of 2018 from *Culicoides* biting midges collected in the Lichuan County, Jiangxi Province of eastern China. The previous survey results of midge species in the northeastern region of Jiangxi Province, including Lichuan County, showed that *Culicoides crakawai*, *Culicoides oxystoma*, and *Culicoides puncticollis* were the main midges in the livestock pens of the region (Liu et al., 2019). The virus can induce cytopathic effects (CPEs) in mammalian cells and death in suckling mice. Sequence determination and analysis of the entire genome of the virus revealed that it was a novel member of the Tete virus serogroup of the *Orthobunyavirus* genus, named Lichuan virus (LICV) based on the area of isolation. This is the first Tete serogroup virus isolated in China. The results are reported below.

## 2 Materials and methods

### 2.1 Specimen collection and preservation

A beef cattle farm in Lichuan County, Jiangxi Province, served as the collection point for the blood-feeding arthropods. Specimens were collected using UV mosquito traps (Wuhan Jixing Environmental Protection Technology Co., Ltd.). The collection time was from 6pm to 6am. In this study, entomologists separated mosquitoes and midges with the naked eye at the specimen collection site, then selected the midge one by one under a stereoscopic microscope. Since morphological identification of midge species requires complex chemical treatment of an individual midge, which cannot be carried out in the field of specimen collection, the midges are referred to only as “ *Culicoides* biting midges “. Collection environment and insect species data were recorded, and the specimens were stored in liquid nitrogen before being transferred to the laboratory for testing (Fu et al., 2017).

### 2.2 Cells

BHK-21 (golden hamster kidney) and C6/36 (*Aedes albopictus*) cells were maintained in our laboratory. BHK-21 cell culture conditions were as follows: 90% Eagle’s medium (laboratory preparation), 7% fetal bovine serum (FBS; Invitrogen), 1% penicillin and streptomycin (100 U/mL), 1% glutamine (30 g/L), and 1% NaHCO_3_. The C6/36 cell culture conditions were as follows: 89% RPMI1640 (Invitrogen), 10% FBS (Invitrogen), and 1% penicillin and streptomycin (100 U/mL). BHK-21 and C6/36 cells were cultured in an incubator at 37°C with 5% CO_2_, and at 28°C, respectively (Cao et al., 2016; Fu et al., 2017).

### 2.3 Virus isolation

1.5 mL of washing solution [90% Eagle’s medium (laboratory preparation), 1% penicillin and streptomycin (100 U/mL), 8% glutamine (30 g/l) and 1% NaHCO3 (75 g/l)] was added to each pool (50–100 mosquitoes or 500 midges) of samples followed by two washes (for three minutes/each wash) for cleaning the body surface of the specimen. Then, 1.5 mL of grinding solution was added (Same with washing solution), and the samples were ground using a glass grinder (Huayi Experimental Equipment Factory) under ice bath conditions. The samples were centrifuged after grinding (20,000 × g, 4°C, 20 min), and 100 μL of the supernatant was inoculated into 80% monolayers of BHK-21 and C6/36 cells on culture plates (24-well plates, Corning Inc.). BHK-21 and C6/36 cells were continuously cultured in an incubator at 37°C with 5% CO_2_, and at 28°C, respectively. Cells were observed for signs of CPEs under a microscope every 12 h. When the cells appeared to be showing CPEs, the virus solution was collected and stored at -80°C until further identification. Specimens that did not show CPEs were blindly passaged for three generations, and those without CPEs were discarded (Cao et al., 2016; Fu et al., 2017).

### 2.4 Detection of virus plaques

BHK-21 cells were transferred onto a 6-well culture plate (Corning Inc.). The next day, once cells had grown into a monolayer covering 80% of the plate, a 10-fold dilution of the virus (10^-1^–10^-6^) was sequentially added to a 6-well culture plate (0.1 mL/well). After adsorption for 1 h in an incubator at 37°C with 5% CO_2_, 1.3% methylcellulose-MEM semi-solid medium (4 mL/well) containing 2% FBS was added to each well. After 72 hours of culture, when a plaque was obvious under the microscope, the medium was discarded, and the plaque was stained with crystal violet. Finally, the number of plaque-forming units (pfu) expressing the virus was calculated (Cao et al., 2016; Feng et al., 2019).

### 2.5 Animal challenge

Suckling mice (2-day-old Kunming mice) were purchased from Beijing Vital River Laboratory Animal Technology Co., Ltd. All mice were raised under sterile conditions at the animal facility of the Chinese Center for Disease Control and Prevention. All animal experiments were conducted in strict compliance with the regulations set by the Animal Ethics Committee of China CDC(NO.2014112509).

Each suckling mice was inoculated with 20 µL of supernatant infected with virus on the fifth passage of BHK-21 cells intracranially. The incidence and mortality of mice were observed and recorded every day. After death, the suckling mice were sacrificed and dissected. The mouse brain was ground, and the ground liquid was centrifuged at 12,000 rpm for 30 min at 4°C, and the supernatant was used to inoculate the next generation of suckling mice. The virus was passaged three times in suckling mice. Animals inoculated with the virus without disease or death were routinely sacrificed after 14 days (Cao et al., 2016).

### 2.6 Viral RNA extraction and cDNA library preparation

The Viral RNA Mini Kit (Qiagen) was used to extract total RNA from the supernatant of virus-infected cells samples according to the manufacturer’s instructions. The extracted RNA was immediately placed in a water bath at 65°C for 10 min and placed on ice for 2 min. Then, 32 μL of RNA was absorbed into the first-strand reaction tube (Ready-To-Go You-Prime First-Strand Beads), which was maintained at room temperature for 1 min before 1 μL of random primers were added (phosphorylated random hexamer primers -pd (N) 6); this was centrifuged immediately and incubated in a water bath at 37°C for 1 h. Moloney Murine Leukemia Virus (M-MuLV) reverse transcriptase was used for reverse transcription. The total volume of the cDNA library was 33 μL, which was used immediately or stored at -80°C for future use (Cao et al., 2016; Fu et al., 2017; Feng et al., 2019).

### 2.7 Identification of viral genome using metagenomic sequencing

Total RNA was extracted from the supernatant on the fifth passage of BHK-21 cells infected with the JXLC1806-2 isolate using the QIAamp Viral RNA Mini Kit (QIAamp, Qiagen, Valencia, CA). A metagenomics shotgun sequencing library was constructed using the TruSeq total RNA library Prep kit (Illumina, San Diego, California) according to the manufacturer’s instructions. The libraries were sequenced on the MiSeq platform (Illumina, San Diego, California) in paired-end 150-bp mode. The sequencing data generated via mNGS were imported to the CLC Genomics Workbench (Version: 20.0.4, Qiagen) and were trimmed based on quality and ambiguity. The clean reads were applied for *de-novo* assembly using the CLC *De Novo* Assembler and contigs of less than 300 bp were discarded from all assembles. The virus-related contigs were extracted based on the taxonomic information of the BLAST hits. To avoid mis-assembly, reads were mapped back to the virus genome and inspected with CLC Genomics Workbench. The viral genome sequences obtained by NGS was used as template for overlapping PCR primers design for Sanger sequencing confirmation.

### 2.8 Viral gene amplification and nucleotide sequence determination

Viral gene amplification (PCR) was carried out in a total volume of 25 μL, and included the cDNA template, GoTaq^®^ Green Master Mix, 2× (Promega, Madison, WI, USA), and 10 μmol/L of upstream and downstream primers. The PCR reaction conditions were pre-denaturation at 95°C, and the amplification conditions of the virus isolate were 94°C 30s, 50°C 30s, 72°C 60s, and a total of 35 cycles were followed by extension at 72°C for 10min. After the PCR reaction was completed, 5 μL of the gene amplification product was detected by 1% agarose gel electrophoresis. The nucleotide sequences of the amplification products were determined by Sanger sequencing (Cao et al., 2016; Fu et al., 2017; Feng et al., 2019). JXLC1806-2 virus gene amplification primers are shown in **Supplementary Material 1**.

### 2.9 5’- and 3’-untranslated regions by 5’/3’ RACE system

Primers were designed based on the obtained partial sequences, and the 5’/3’ RACE system for rapid amplification of cDNA ends (Invitrogen) was used to amplify the gene sequences of the 5’ untranslated region (UTR) and 3’ UTR. For the 3’ UTR without a poly(A) tail, the Poly (A) Polymerase Tailing Kit (Epicentre) was used to add the A-tail prior to amplification. The amplified products were detected by 1% agarose gel electrophoresis, purified using a QIAquick Gel Extraction Kit (Qiagen), cloned into the pGEM-T Easy vector (Promega), and 10 clones of each product for each region were sequenced by the Sanger method (Wang et al., 2015; Cao et al., 2016).

### 2.10 Nucleotide sequence analysis

The sequences of representative strains of *Orthobunyavirus* isolated from different hosts and countries for different years were downloaded from GenBank. The virus strain information used in the analysis is shown in **Supplementary Material 2**. The nucleotide sequences were subjected to a Blast (NCBI) alignment. The Seqman software (DNAStar, Madison, WI, USA) was used to splice the amplified sequence of the complete set of primers, and splicing errors were found to make the sequence more accurate.; BioEdit software (Version 7.0, Thomas) was used for nucleotide multiple sequence alignment; MEGA6.0 software was used for neighbor-joining phylogenetic analysis with a bootstrap value of 1000; homology analysis of nucleotide and amino acid sequences was performed using MegAlign (DNAStar, Madison, WI, USA) (Wang et al., 2015; Cao et al., 2016; Fu et al., 2017).

## 3 Results

### 3.1 Collection of blood-feeding arthropods specimens

In July 2018, 12,400 *Culicoides* biting midges, two genera, and two species of mosquitoes were collected in Lichuan County, Jiangxi Province, which contained 270 *Culex tritaeniorhynchus* and 230 *Anopheles sinensis* (**Table 1**).

**Table 1:** Collection of blood-feeding arthropods specimens in Lichuan County, Jiangxi Province in 2018.

Date of specimen collection	Breeding place^*^	Number of mosquitoes		Number of *Culicoides* biting midges	Total
		Culex tritaeniorhynchus	Anopheles sinensis		
7.8	beef cattle farm 1	30	60	2500	2590
7.8	beef cattle farm 2	140	70	3500	3710
7.8	beef cattle farm 3	100	100	6400	6500
Total		270	230	12400	12900

*The collections were from three different beef cattle farms, and the distance between each farm was approximately 300–500 m. The JXLC1806-2 virus was isolated from Culicoides biting midges collected from beef cattle farm 2

### 3.2 Virus isolation

**Figure 1:**
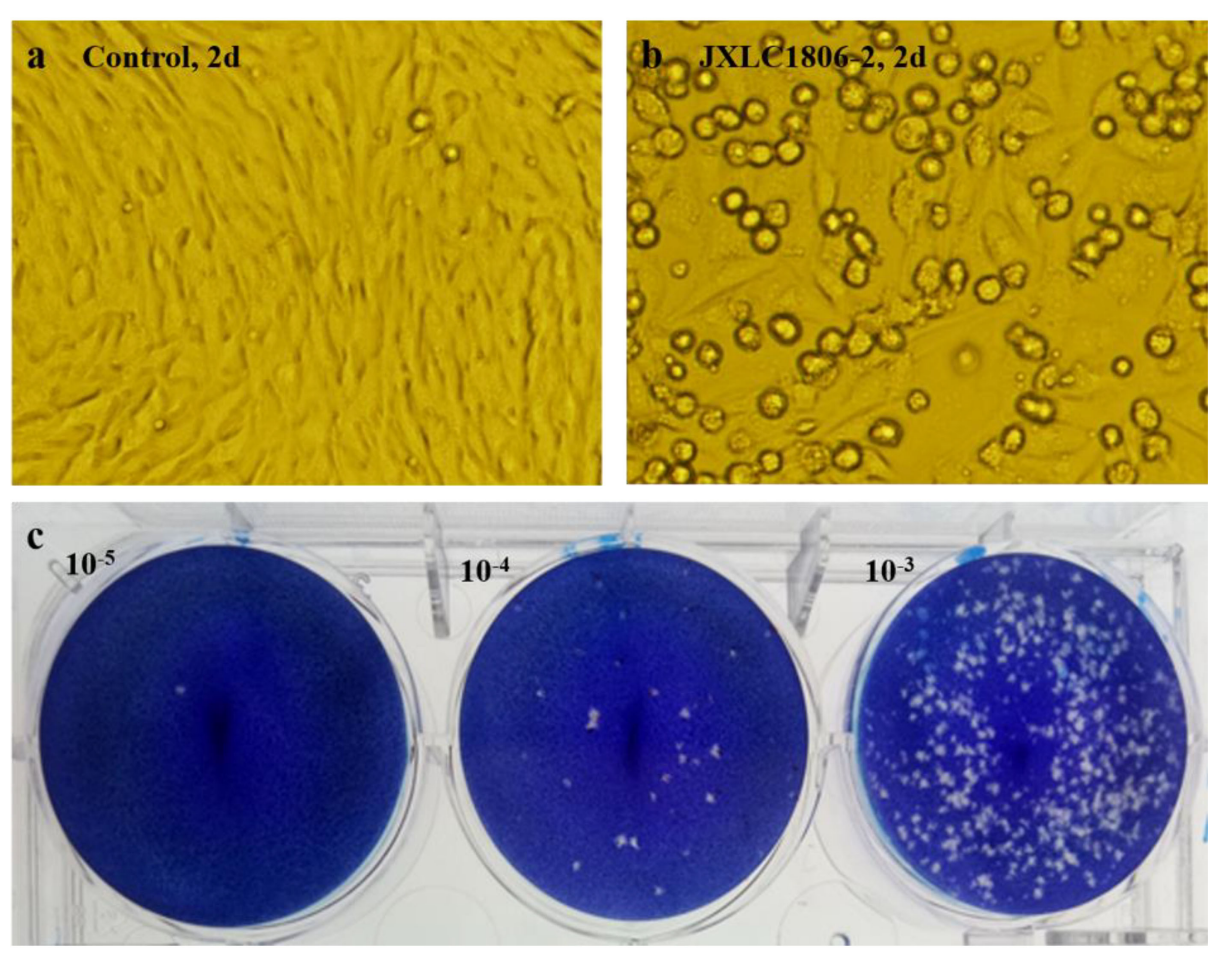
CPEs and plaques of the JXLC1806-2 virus in BHK-21 cells. **(A, B)** show the morphologies of normal BHK-21 cells and BHK-21 cells cultured to 48h after inoculation with the JXLC1806-2 virus, respectively. Magnification, 200x. Figure **(C)** shows the plaque morphology of the JXLC1806-2 virus in BHK cells.

The collected blood-feeding arthropods specimens were pooled and ground in the laboratory. There were six pools of mosquitoes and 21 pools of midges. The ground supernatants were inoculated into BHK-21 and C6/36 cells, and the culture was maintained, with the CPEs status observed on a daily basis. After three consecutive generations of cultivation, only JXLC1806-2 sample caused CPEs in the second and third generation BHK-21 cells, these effects included cell shrinkage, shedding, and reduction of adherent cells (**Figure 1**). No CPEs was observed in BHK-21 cells in the remaining 26 sample pools. The CPEs time of JXLC1806-2 on second and third generations BHK-21 was 72 h and 48 h, respectively. After 5 passages of JXLC1806-2 virus in BHK-21 cells, the time of CPEs was stable at 48h. When 27 griding supernatant sample pools, including JXLC1806-2, were continuously cultured in C6/36 cells for three generations, no CPEs was observed. And no replication was detected by RT-PCR in the supernatant of three consecutive generations of C6/36 cells for JXLC1806-2.

### 3.3 Viral plaque and titer

The JXLC1806-2 virus isolate could form virus plaques with regular plaque shapes and clear edges in BHK-21 cells. The diameter of the plaques was 1.19 mm (1.45 ± 0.30 mm, n = 10, 3d) (**Figure 1**). The virus titer determined by the plaque formation method showed that the JXLC1806-2 virus titer of the fifth passage of BHK cells was 1 × 10^5.6^ pfu/mL.

### 3.4 Animal pathogenicity

**Figure 2:**
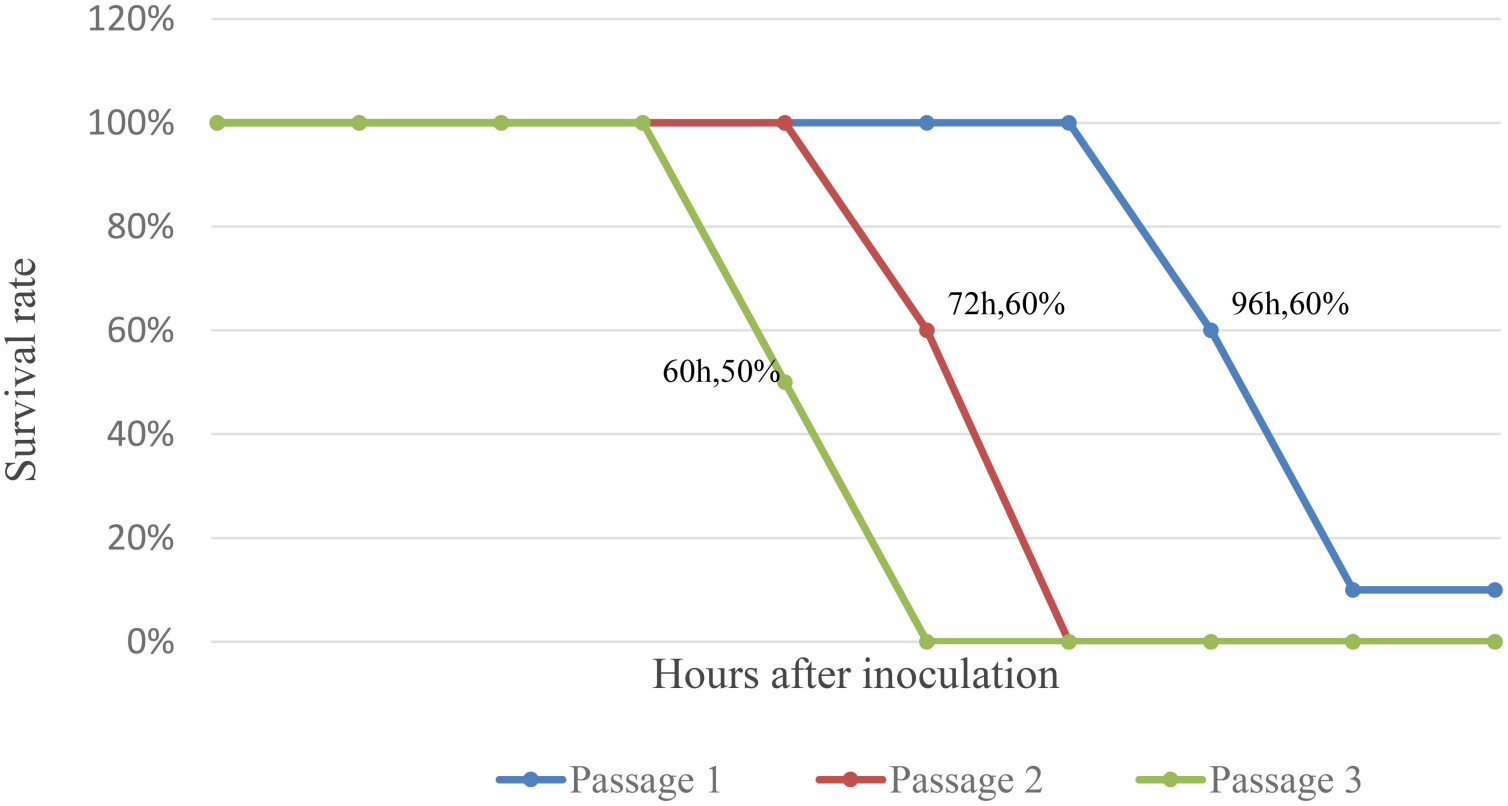
Survival curve of Suckling mice after inoculation with JXLC1806.

The results showed that four of ten mice inoculated in the first generation gradually developed illness (no food intake, stiff limbs, and negative righting reflex) and died within 96 h of inoculation, five mice died within 108 h of inoculation, with the remaining one mice being maintained to 14 days without illness or death; Two of five mice inoculated in the second generation died 72 h after inoculation, with the remaining three mice died 84 h after inoculation; Three of six mice inoculated in the third generation died 60 h after inoculation, with the remaining three mice died 72 h after inoculation (**Figure 2**).

### 3.5 Molecular biology characteristics of the virus

#### 3.5.1 Viral gene sequence determination and analysis

In this study, the partial sequence of virus isolate was first obtained from the supernatant infected with virus on the fifth passage of BHK-21 cells sample through second-generation sequencing. The primers were designed (**Supplementary Material 1**) to amplify and Sanger sequencing of the nucleotide sequence of the whole genome of the JXLC1806-2 virus. Nucleotide sequence analysis showed that the JXLC1806-2 virus genome was composed of S segment (GenBank number: MT198371), M segment (GenBank number: MT198372), and L segment (GenBank number: MT198373) segments, and the nucleotide and amino acid sequence lengths of the open reading frames of the S, M, and L segments were 774 nt (258 aa), 4299 nt (1433 aa), and 6840 nt (2280 aa), respectively. A comparison of the nucleotide and amino acid sequences of the segments of the open reading frame of the JXLC1806-2 virus with the corresponding segments of the Tete serogroup virus of the *Orthobunyavirus* genus showed that the JXLC1806-2 virus had 66.5% (Batama virus) to 75.0% (Matruh virus) of nucleotide identity, and 69.8% (Batama virus) to 77.2% (Bahig virus) of amino acid similarity with the S segment of other viruses in this group; 70.2% (Tete virus) to 78.0% (Matruh virus), and 75.1% (Tete virus) to 86.2% (Marruh virus) with the M segment; and 74.5% (I612045 virus) to 80.4% (Matruh virus), and 81.6% (I612045 virus) to 92.2% (Matruh virus) with the L segment. A comparison of JXLC1806-2 or the Tete virus with the Oyo virus outsidethe Tete group revealed differences in the lengths of nucleotide and amino acid sequences of the three segments as well as lower homology than other viruses in the Tete serogroup (**Table 2**).

**Table 2:** JXLC1806-2 virus genome open reading frame (ORF) nucleotide amino acid homology.

Virus	S		M		L	
	nt (%)	aa (%)	nt (%)	aa (%)	nt (%)	aa (%)
JXLC1806-2	774	258	4299	1433	6840	2280
Tete virus	774 (69.3%)	258 (71.0%)	4299 (70.2%)	1432 (75.1%)	6843 (75.1%)	2281 (82.6%)
Matruh virus	774 (75.0%)	258 (76.8%)	4299 (78.0%)	1433 (86.2%)	6840 (80.4%)	2280 (92.2%)
Bahig virus	774 (74.8%)	258 (77.2%)	4299 (77.2%)	1433 (85.8%)	6840 (80.1%)	2280 (91.8%)
I612045	771 (70.1%)	257 (72.4%)	4296 (71.4%)	1432 (76.6%)	6840 (74.5%)	2280 (81.6%)
Batama virus	774 (66.5%)	258 (69.8%)	–	–	–	–
Oyo virus	765 (62.5%)	255 (60.0%)	4293 (53.5%)	1431 (40.7%)	6720 (62.5%)	2240 (59.1%)

nt indicates nucleotide.

aa indicates amino acid.

- indicates a missing reference nucleotide sequence.

#### 3.5.2 Molecular genetic evolution of the viral genome

**Figure 3:**
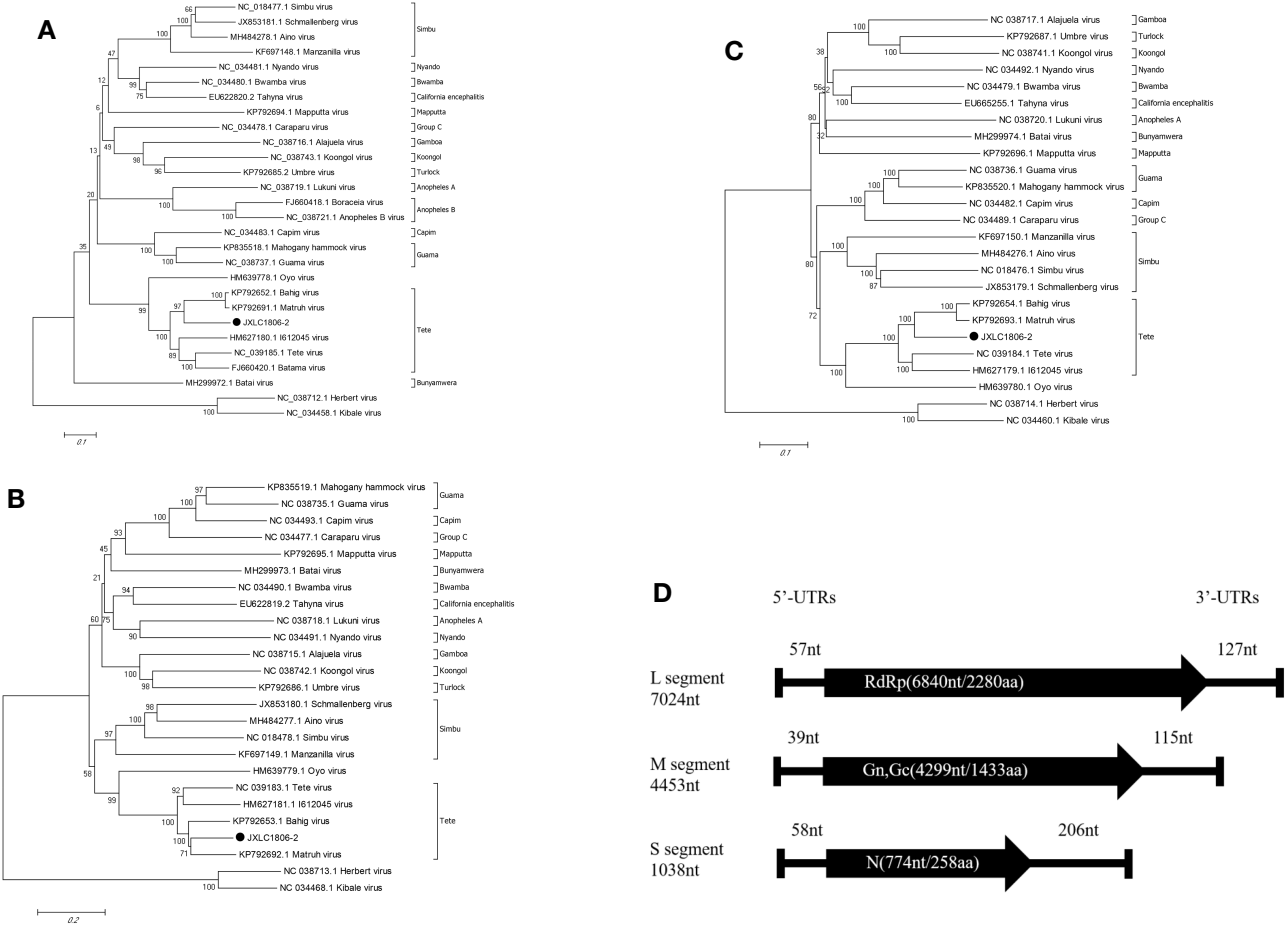
Phylogenetic tree constructed based on the nucleotide sequence of *Orthobunyavirus*. **(A–C)** are phylogenetic trees constructed from the nucleotide sequences of the S, M, and L segments of each of the serogroup viruses of the *Orthobunyavirus* genus. **(D)** is a schematic representation of the JXLC1806-2 virus genome. MEGA6.0 software was used for neighbor-joining phylogenetic analysis with a bootstrap value of 1000. The black padding shows the JXLC1806-2 virus strain isolated in this study. **Figure 2D** is a schematic representation of the JXLC1806-2 virus genome.

Viral genetic evolution analysis of the nucleotide sequences of the represented virus strains of each of the serogroup viruses of the *Orthobunyavirus* genus showed that the nucleotide sequence of the open reading frame of the JXLC1806-2 virus (S, M, or L segments), belonged to the *Orthobunyavirus* Tete serogroup, and the JXLC1806-2 virus was independent of the Tete viruses, Matruh virus, and Bahig virus, which formed a separate evolutionary branch. In this paper, the ML method (**Supplementary Material 3**) is used for the phylogenetic analysis, and the results are the same as NJ method (**Figure 3**). The amino acid homology of N protein between JXLC1806-2 virus and Tete virus serogroup virus was 69.8%-77.2%, less than 90%, suggesting that JXLC1806-2 is a novel member of the Tete serogroup (**Figure 3**).

#### 3.5.3 Characteristics of the 5’ UTR and 3’ UTR of the JXLC1806-2 virus

The 5’ and 3’ RACE methods were used to obtain the nucleotide sequences of the 5’ and 3’ non-coding regions located on either side of the coding regions of the S, M, and L segments of the JXLC1806-2 virus. The lengths of the 5’ UTR regions of the three segments (S, M, and L) of the JXLC1806-2 virus strain were 58, 39, and 57 bp, respectively, and the lengths of the 3’ UTR regions of the three segments were 206, 115, and 127 bp, respectively. The 5’ and 3’ ends of the three segments of the S, M, and L segments were all reverse complementary sequences of 8 nucleotides (5’-AGTAGTGT … ACACTACT -3’) (**Table 3**, **Supplementary Material 4**).

**Table 3:** Nucleotide sequence characteristics of the non-coding region of the JXLC1806-2 virus.

	S segment		M segment		L segment	
Virus	5’UTR	3’UTR	5’UTR	3’UTR	5’UTR	3’UTR
JXLC1806-2	58^*^ AGTAGTGTACC	206 TCATCACACGA	39 AGTAGTGTACT	115 TCATCACACGA	57 AGTAGTGTGCT	127 TCATCACACGG
Tete virus	73 AGTAGTGTACT	172 TCATCACATGA	\	\	\	\
Batma virus	73 AGTAGTGTACT	172 TCATCACACGA	\	\	\	\
Bahig virus	\	\	39 AGTAGTGTACT	\	58 AGTAGTGTACC	\
Matruh virus	\	\	\	\	58 AGTAGTGTACC	\
I612045	67 AGTAGTGTACT	179 TCATCACACGA	41 GAGTAGTGTACT	142 ATCATCACACGA	68 AGTAGTGTGCT	\

*represents length of the UTR nucleotide sequence

AGTAGTGTACC represents a conserved sequence and a variation site

\ represents an unavailable sequence

To understand the differences between the nucleotide sequences of the UTR regions of the three segments of the JXLC1806-2 virus and the Tete serogroup virus, the JXLC1806-2 virus UTR sequence was compared with that of Tete group viruses (Tete, Bahig, Matruh, Batama, and I612045 viruses). The results showed that the UTRs on both sides of the three segments of the JXLC1806-2 virus were consistent with the nucleotide sequences of other viruses in the Tete group. There were only three nucleotide variations at the 9^th^ position of the 5’ UTR (conserved region of the virus non-coding region), the 11^th^ position of the 3’ UTR of the virus L segment, and the 11^th^ position of the S segment 5’ UTR (**Table 3**, **Supplementary Material 4**).

## 4 Discussion

In this paper, a *Culicoides* biting midges specimen collected in the nature of Jiangxi Province in southeastern China was isolated from a virus isolate (JXLC1806-2) that can cause cytopathic effects in mammalian cell, which can cause morbidity and death in suckling mice. Sequencing and analysis of the entire genome of the virus showed that JXLC1806-2 virus was RNA virus with three segments. The results of virus gene sequence homology and molecular genetic evolution analysis showed that the virus is a new member of the Tete virus group in the *Orthobunyavirus* genus, named Lichuan virus (LICV). This is the first time that Tete virus group has been isolated from *Culicoides* biting midges specimens collected in nature in China.

### 4.1 JXLC1806-2 virus is a novel member of the Tete virus group

As mentioned earlier, the *Orthobunyavirus* contained S, M, and L segmentss, and the S segment was generally 1000 nt in length and encoded two proteins (N and Ns protein) (Shchetinin et al., 2015; Lefkowitz et al., 2017). However, recent results have shown that the S segment open reading frame sequences of all viruses in the Tete serogroup lacked the Ns protein sequence, the Tete virus S segment open reading frame of the *Orthobunyavirus* was 774 nt in length and encoded a 258-aa N protein (Mohamed et al., 2017). Therefore, the S gene nucleotide sequence only encoded the N gene and lacked the Ns protein nucleotide sequence, which is characteristic of Tete serogroup viruses (Mohamed et al., 2017; Vuren et al., 2017). The S segment of the JXLC1806-2 virus was 774 nt in length (encoding 258 aa), which was the same as the nucleotide and amino acid length of the S segmentof the Tete virus (**Table 2**), suggesting that the JXLC1806-2 virus S segment exhibited characteristics of a Tete serogroup virus, both in terms of nucleotide sequence length and open reading frame sequence.

Evolutionary molecular genetic analysis based on the nucleotide sequence of the virus S segment revealed that the Tete serogroup virus was significantly different from the OYO virus, which was placed outside of the serogroup, and was divided into two large evolutionary branches; the first branch contained the Tete, Batama, and I612045 viruses (the I612045 virus was independent of the first two viruses), and the second branch contained the Matruh and Bahig viruses. Evolutionary analysis of the JXLC1806-2 virus S segment and the five Tete serogroup viruses showed that all six of these viruses were divided into two evolutionary branches. The JXLC1806-2 virus was independent of the Matruh and Bahig viruses and formed a separate evolutionary branch (**Figure 2**). The amino acid homology of N protein between JXLC1806-2 virus and Tete virus serogroup virus was 69.8%-77.2%, less than 90%. From the evolutionary molecular genetic results and homology analysis results, it can be seen that the JXLC1806-2 virus represents a new species of Tete virus group as part of *Orthobunyavirus*.

### 4.2 Tete serogroup virus vector and viruses isolated from midges in China

The Tete serogroup which includes the Tete (Taylor, 1967; Shchetinin et al., 2015), Bahig (Converse et al., 1974), Matruh (Balducci et al., 1973), I612045 (NCBI, 2012), Batama (Karabatsos, 1978), and Weldona viruses (Calisher et al., 1990) were isolated from birds, ticks and midges, Thus, birds may be the natural hosts of Tete serogroup viruses, and ticks and midges may be the vectors of these viruses. In this study, the JXLC1806-2 virus was isolated from *Culicoides* biting midges, suggesting that midges were vectors for the Tete serogroup virus. A number of viruses that cause human and animal diseases have been isolated from midges collected from across China (Liang et al., 2017), including the Banna virus of the Seadornavirus genus of Reoviridae (Song et al., 2017), Tibetan circovirus of the Orbivirus genus (Lei et al., 2015; Wang et al., 2017), Getah virus of the alphavirus (Dong et al., 2017), and Japanese encephalitis virus of the flavivirus (Zheng et al., 2012). In this study, the JXLC180602 virus was the first isolate of the *Orthobunyavirus* Tete serogroup virus isolated from *Culicoides* biting midges specimens, further suggesting that Chinese midges can carry a variety of arboviruses. This makes our study of great significance for public health, as it will increase our ability to identify and isolate viruses present in midges.

### 4.3 The collection sites of JXLC1806-2 virus

Lichuan County, located in the eastern part of Jiangxi Province, has a mild climate, sufficient sunshine, abundant rainfall, and four distinct seasons, and has a mid-subtropical humid climate (People’s government of lichuan county, 2018), making it suitable for breeding various blood-feeding arthropods. Survey results of midge species in livestock pens in the northeastern region of Jiangxi Province, including Lichuan County, showed that *Culicoides crakawai*, *Culicoides oxystoma*, and *Culicoides puncticollis* were the main local midges in the livestock pens (Liu et al., 2019). However, there were differences in the proportions of these three midges in the different livestock pens. Among them, *Culicoides crakawai* in duck and hen houses accounted for 96.51% and 72.87%, respectively, while 82.98% of the total midges from the piggery were *Culicoides oxystoma*, and *Culicoides puncticollis* accounted for 59.74% in the sheep pens. Midges collected from the cattle farm were identified as *Culicoides crakawai* (53.31%), *Culicoides oxystoma* (12.16%), and *Culicoides puncticollis* (7.89%). *Culicoides crakawai* was dominant in the local duck and hen houses, *Culicoides oxystoma* dominated in the piggery, and *Culicoides puncticollis* dominated in the sheep pens. The cattle farm had various types of midges (Liu et al., 2019). The midges collected in Lichuan County, Jiangxi Province, came from a beef cattle farm that covered an area of approximately 30 hectares. The farm was divided into three areas that were separated by roads. Each area contained approximately 100 heads of beef cattle, and the farm was approximately 10 km away from the village. We collected 3,500 midges from the second breeding ground (**Table 1**), and these were separated into eight pools for virus isolation. Among them, the JXCL1806-2 pool of midge grinding liquid contained the JXLC1806-2 virus, which was isolated in BHK cells. For morphological identification of the midges, each midge body was dissected, fixed, covered with a glass slide, and viewed under a microscope. However, following this process, the midge specimens were no longer suitable for virus isolation. Therefore, the JXLC1806-2 midge pool was identified based on morphology alone, and the midge species could not be identified. In the future, molecular biology methods will be used (via the COI gene) to identify the midge species prior to virus isolation. This will enable us to clarify the specific species of midges carrying the viruses.

## 5 Conclusion

JXLC1806-2 is the first Tete serogroup virus isolated from *Culicoides* biting midges in China and represents a novel member of the Tete serogroup. The JXLC1806-2 virus not only causes CPEs in mammalian cells but can also cause morbidity and death in suckling mice, but it does not cause CPEs in C6/36cells, and no replication was detected by RT-PCR. Because the virus was isolated from *Culicoides* biting midges in cattle farms, it is of considerable significance to public health and will enhance our ability to detect and monitor JXLC1806-2 virus infection in local cattle herds. It will also enable us to investigate whether the virus can cause infections and diseases in other local animals.

## Data availability statement

The datasets presented in this study can be found in online repositories. The names of the repository/repositories and accession number(s) can be found in the article/**Supplementary Material.**

## Ethics statement

The animal study was approved by The meeting of ethics committee of national institute for viral disease control and prevention, China CDC. The study was conducted in accordance with the local legislation and institutional requirements.

## Author contributions

QY, RC, XX and GL involved in drafting the manuscript. QY, RC, XX and GL involved in revising the manuscript. RC, ZX, JW, SF, HX, SZ YH, FL and SX participated in the acquisition of data. QY, RC, XX, ZX, JW, SF, XL and GL participated in the analysis and interpretation of data. SF, HW, BW and GL supported funding for the study. HW, BW and GL contributed to conception and design of the manuscript. All authors contributed to the article and approved the submitted version.
