# Supplementary material for: Isolation and identification of Tete virus group (Peribunyaviridae: Orthobunyavirus) from Culicoides biting midges collected in Lichuan County, China
Source: Front Cell Infect Microbiol. 2023 Oct 31;13:1193184. doi: 10.3389/fcimb.2023.1193184 (PMC10644344; doi:10.3389/fcimb.2023.1193184)
Supplement: Supplementary file 1 [file DataSheet_1.docx]

Supplementary Material

Isolation and Identification of Tete virus group (Peribunyaviridae: Orthobunyavirus) from midges collected in Lichuan County, China

Qikai Yin^†^, Rui Cheng^†^, Xiuyan Xu^†^, Ziqian Xu^†^, Jing Wang, Shihong Fu, Hongbin Xu，Shaozai Zhang，Ying He，Fan Li，Songtao Xu，Xiaoqing Lu，Huanyu Wang*，Bin Wang*，Guodong Liang*

*** Correspondence:** Huanyu Wang; Bin Wang; Guodong Liang: [wanghy@ivdc.chinacdc.cn](mailto:wanghy@ivdc.chinacdc.cn); [wangbin532@126.com](mailto:wangbin532@126.com); [gdliang@hotmail.com](mailto:gdliang@hotmail.com)

# Supplementary Material 1. JXLC1806-2 virus gene amplification primers.

| Primer | Sequence of primers(5，—3，) | Annealing temperature（℃） |
| --- | --- | --- |
| S gene |  |  |
| S-1-700F | AGTAGTGTACCCCACAAAATACAAAATCGT | 55 |
| S-1-700R | TGCACCGGTTGTCAACATCT |  |
| S-5-450F | CCCACAAAATACAAAATCG | 50 |
| S-5-450R | GTCAGGAGGACAGCATAAG |  |
| S-466-1015F | CCCAAAGCAAAAGGACACCG | 53 |
| S-466-1015R | AGTAGTGTGCTCCACAAATACAT |  |
| M gene |  |  |
| M-1-562F | AGTAGTGTACTACCATTTAGGAAAACGTGTT | 55 |
| M-1-562R | ACGCATGGATTGGCTTCTGA |  |
| M-5-1079F | TGTACTACCATTTAAGAAAACG | 50 |
| M-5-1079R | TCACCAATCTCTGTCAAACTAT |  |
| M-680-2035F | TGTGACATAATCTGCTGCTC | 50 |
| M-680-2035R | ATACTTGTAGCCAACCCTCT |  |
| M-1838-3455F | TAATCTGTGTGCTTACTACCC | 53 |
| M-1838-3455R | GACCACTACATCTTTTCTTTG |  |
| M-1908-2988F | GGGAACAACTGAGACATTT | 50 |
| M-1908-2988R | TTTGAACCCATTAGCCATT |  |
| M-2818-3600F | ACTACACCTGTTCAAGCCGAT | 50 |
| M-2818-3600R | AAAGCACTTCCTGACCACTAC |  |
| M-3302-4288F | ACCTATGCGTAACTATGCC | 50 |
| M-3302-4288R | CCTTTCTGTCGAGTTTTTG |  |
| M-4059-4453F | AGAGGTTCTGGATTTGGCCC | 54 |
| M-4059-4453R | AGTAGTGTGCTACCATATAAGAATTATCG |  |
| L gene |  |  |
| L-1-712F | AGTAGTGTGCTCCAGGGTTTCGTATTAC | 56 |
| L-1-712R | GGGCTTCATGTTTTGCTCCCA |  |
| L-1-1861F | AGTAGTGTACCCCTGGGTTTCGTA | 53 |
| L-1-1861R | CTGACATCTTTCCTTATCCAATCT |  |
| L-572-1245F | CCTTGGGTGAATGAAGAAAC | 50 |
| L-572-1245R | AGAAATCCGACCTTGAGTAT |  |
| L-1644-2913F | TTAGAGTTGTATGTTGTGCC | 50 |
| L-1644-2913R | CAGTCTTTTGTCCTTTATTG |  |
| L-2758-4463F | GTACATAGACGTCCAATCAACG | 53 |
| L-2758-4463R | TTCTTCTGTGGTCTCACCTTTT |  |
| L-4174-5054F | TGGAACTTGGAACAACTAAC | 50 |
| L-4174-5054R | CTCTTGCTCTCATCATCATT |  |
| L-4824-5667F | GAGAGGACTGGAATGTCAAGCAAT | 53 |
| L-4824-5667R | CCAATCTATCATCTTCGCCTGTAA |  |
| L-5238-6715F | ATATTTCAGACCAAGGTGCT | 50 |
| L-5238-6715R | TCGGAATGTGGAGATAGATG |  |
| L-6564-6950F | ATTACCACCTATTTGAGATGC | 50 |
| L-6564-6950R | AGCTTAGCTGACTAATTCACC |  |
| L-6863-7024F | AGGTGGAAGGTGGGAAATCG | 54 |
| L-6863-7024R | AGTAGTGTGCCCCTGGATGTAG |  |

# Supplementary Material 2. Virus sequence information used in this study

| .Virus | | Strains | Year | Country | Host | GenBank number | | |
| --- | --- | --- | --- | --- | --- | --- | --- | --- |
|  |  |  |  |  | S | M | L |  |
| LiChuan virus | | JXLC1806-2 | 2018 | China | Midge | MT198371 | MT198372 | MT198373 |
| Tete virus | | SAAn 3518 | 1959 | South Africa | Ploceus cucullatus | NC_039185.1 | NC_039183.1 | NC_039184.1 |
| Matruh virus | | An 1047-61 | 1961 | Egypt | Sylvia curraca | KP792691.1 | KP792692.1 | KP792693.1 |
| Bahig virus | | EgB 90 | 1966 | Egypt | Oriolus oriolus | KP792652.1 | KP792653.1 | KP792654.1 |
| Batama virus | | AnB1292 | - | Central African Republic | Tick | FJ660420.1 | - | - |
| I612045 virus | | - | 1961 | India | - | HM627180.1 | HM627181.1 | HM627179.1 |
| Oyo virus | |  | 1964 | Nigeria | Armigeres subalbatus | HM639778.1 | HM639779.1 | HM639780.1 |
| Capim virus | | BeAn 8582 | 1958 | Brazil | Caluromys philander (woolly opossum) | NC_034483.1 | NC_034493.1 | NC_034482.1 |
| Mahogany hammock virus | | FE4-2s | 1964 | USA | Culex (Melanoconion) sp. | KP835518.1 | KP835519.1 | KP835520.1 |
| Guama virus | | BeAn 277 | 1955 | Brazil | Cebus apella | NC_038737.1 | NC_038735.1 | NC_038736.1 |
| Caraparu virus | | BeAn3994 | 1956 | Brazil | Cebus apella | NC_034478.1 | NC_034477.1 | NC_034489.1 |
| Koongol virus | | MRM31 | 1960 | Australia | Culex annulirostris | NC_038741.1 | NC_038742.1 | NC_038743.1 |
| Umbre virus | | IG1424 | 1955 | India | Culex bitaeniorhynchus | KP792685.2 | KP792686.2 | KP792687.2 |
| Alajuela virus | | MARU 11079 | 1963 | Panama | Aedeomyia squamipennis | NC_038716.1 | NC_038715.1 | NC_038717.1 |
| Bwamba virus | | M459 | 1937 | Uganda | Homo sapiens | LC223132 |  |  |
| Tahyna virus | | XJ0625 | 2006 | China | Culex sp. | EU622820.2 | EU622819.2 | EU665255.1 |
| Batai virus | | PV424 | 2016 | Germany | Phoca vitulina | MH299972.1 | MH299973.1 | MH299974.1 |
| Lukuni virus | | TRVL 10076 | 1955 | Trinidad and Tobago | Ochlerotatus scapularis | NC_038719.1 | NC_038718.1 | NC_038720.1 |
| Boraceia virus | | Ar395 | 1962 | Brazil | Mosquito | FJ660418.1 | - | - |
| Anopheles B virus | | - |  | Colombia | - | NC_038721.1 | - | - |
| Nyando virus | | MP401 | 1959 | Kenya | Anopheles funestus | NC_034481.1 | NC_034491.1 | NC_034492.1 |
| Mapputta virus | | MRM186 | 1960 | Australia | Anopheles meraukensis | KP792694.1 | KP792695.1 | KP792696.1 |
| Akabane virus | | DHL10M110 | 2010 | China： | Anopheles vagus | KY284023.1 | KY284022.1 | KY284021.1 |
| Simbu virus | | SA Ar 53 | - | - | - | NC_018477.1 | NC_018478.1 | NC_018476.1 |
| Sathuperi virus | | Germany | 2011 | Germany | - | JX853181.1 | JX853180.1 | JX853179.1 |
| Manzanilla virus | | TRVL 3586 | 1954 | Trinidad and Tobago | Alouatta seniculus | KF697148.1 | KF697149.1 | KF697150.1 |
| Herbert virus | | F23/CI/2004 | 2004 | Cote d'Ivoire | Culex nebulosus | NC_038712.1 | NC_038713.1 | NC_038714.1 |
| Kibale virus | | P05/UG/2008 | 2008 | Uganda | Culex simpliciforceps | NC_034458.1 | NC_034468.1 | NC_034460.1 |
| Tai virus | | F47/CI/2004 | 2004 | Cote d'Ivoire | Culicidae sp. | NC_034457.1 | NC_034461.1 | NC_034459.1 |
| Aino virus | | JaNAr 28 | 1964 | Japan | Culex tritaeniorhynchus | MH484278.1 | MH484277.1 | MH484276.1 |

# Supplementary Material 3.

Molecular Phylogenetic analysis by Maximum Likelihood method.

a


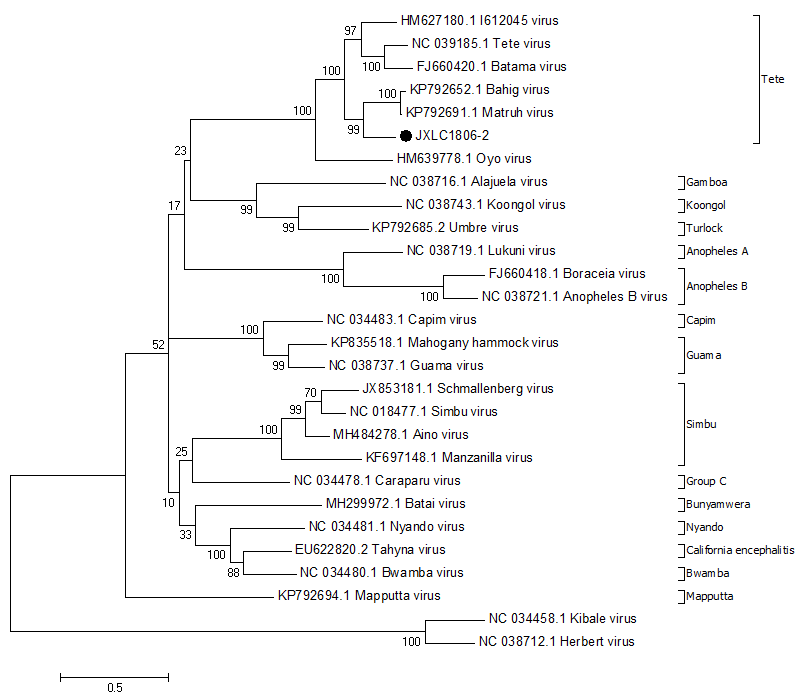


b


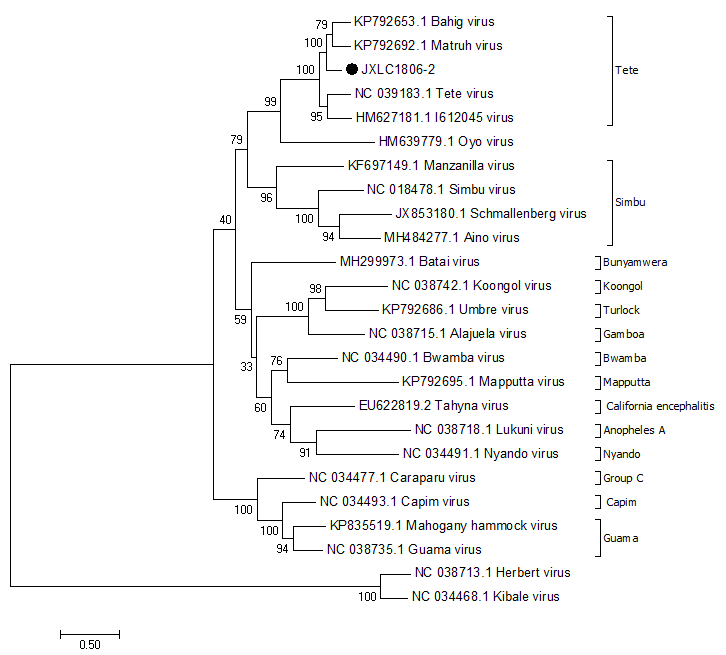


c


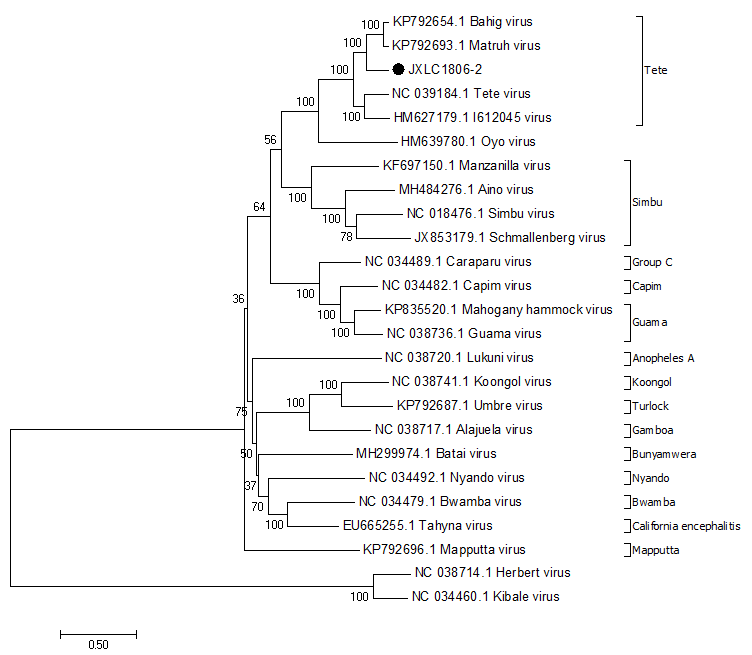


Supplementary Figure 3 a, b, and c are phylogenetic trees constructed from the nucleotide sequences of the S, M, and L segments of each of the serogroup viruses of the Orthobunyavirus genus by Maximum Likelihood method. The black padding shows the JXLC1806-2 virus strain isolated in this study.

# Supplementary Material 4. Comparison of the nucleotide sequences of the 5' and 3' ends of the JXLC1806-2 virus with other Tete serogroup viruses.

* 20 * 40 * 60 * 80 * 100 *

**5’L_JXLC1806-2**  : -AGTAGTGTGCTCCAGGGTTTCGTATTACTTTTAAAACAATTTTCTTTATCTGAAAAA---------------------------------------------------------- : 57
5’L_Bahig_virus : -AGTAGTGTACCCCTGGGTTTCGTATTACCAATAAAACAAATTTCTTTACTTGTGAAAA--------------------------------------------------------- : 58
5’L_Matruh_virus : -AGTAGTGTACCCCTGGGTTTCGTATTACCAATAAAACAAATTTCTTTACTTGTAAAAA--------------------------------------------------------- : 58
5’L_I612045_ : -AGTAGTGTGCTCCACGGGTTTCATATTATAATAAATAATCAATCAATCTCAATTCTTAACATTCAATC----------------------------------------------- : 68

**5’M_JXLC1806-2**  : -AGTAGTGTACTACCATTTAGGAAAACGTGTTATTACAAA---------------------------------------------------------------------------- : 39

5’M_Bahig_virus : -AGTAGTGTACTACCATTTAAGAAAACGTTTTATTTCAAA---------------------------------------------------------------------------- : 39
5’M_I612045_ : GAGTAGTGTACTACCATTTAAGAAAACGCTTTATTAGCAAA--------------------------------------------------------------------------- : 41

**5’S_JXLC1806-2**  : -AGTAGTGTACCCCACAAAATACAAAATCGTTACAAATCATTCAAAACTTTGACTTAAA--------------------------------------------------------- : 58

5’S_Tete_virus : -AGTAGTGTACTCCACTGGATACAAAATCGTTAATACTGAGAATTTATAGATTGCCAATAGCAAATCATACAAA------------------------------------------ : 73
5’S_Batama_virus : -AGTAGTGTACTCCACAAAATACAAAATCGTTAATACTGAGAATTTATAGATTGCATATAGCAAACCATACAAA------------------------------------------ : 73
5’S_I612045_ : -AGTAGTGTACTCCACAAAATACAAAATCGTTAGAACCTTGAATTCTTATATCTCTTAGATATTCAAC------------------------------------------------ : 67

* 20 * 40 * 60 * 80 * 100 *
**3’L_JXLC1806-2**  : --------------------------------------------------------GTAGAGAGGAGTGAGGACTGGAAGAAACCTACTTTTGTAGGTGAACTATTCAGCATAATTG : 61

**3’M_JXLC1806-2**  : --------------------------------------------------------------------AATCCGGCCTCTATATAGAAAAGTTAGCAACCCTTTTGGGTTGCTAACG : 49
3’M_I612045_ : ---------------------------------------GCTGTACAATTAGTTTTAGGTAGTTAGCAACCCTTTGGGTTGCTAACAAAAGCAACAGCTATAGCTACGTTATTATAG : 78

**3’S_JXLC1806-2**  : ATTTTCCCTCTTTAGGGATAGGGGTTGGTATTTATACCAACCCCGACATGCAGCAGCAATCAAAATATCATACTAAATGTGGTGGGTGGTTGGGGCATGAAGCTGCAATAGCTTATT : 117

3’S_Tete_virus : --------ACAATTCTCTTTTAAGAGATAGGGGTTGGTATTTATATACCAACTCCGACATGTAAACAGCTTAAGTAAGCTGTAATATGAGGTGGGTGGTTGGGGCAAGGTTCAGATC : 109
3’S_Batama_virus : --------ATAATTCTCTTTTAAGAGATAGGGGTTGGTATATTTACCAACCCCGACATGTAAACAGCTAAATTAAGCTGCAATATGTGGTGGGTGGTTGGGGCAAGATTCAGATCAA : 109
3’S_I612045_ : -ATTTCTCTTTTAAGAGATAGGGGTTGGTATTTATACCAACCCCGACATGTAAACAGCAGATATAATATTATTAAGCTTTGAAAGTGAGGTGGGTGGTTGGGGCTAGTATCCAGCCT : 116

120 * 140 * 160 * 180
**3’L_JXLC1806-2**  : ATTTTAACTTAAAATATTAAATTCTAAAATAAATACATATACTACATCCAGGGGCACACTACT- : 124

**3’M_JXLC1806-2** : AAAGCAACTGCAGTACTACTCTCTTTCTTCTTGACGATAATTCTTATATGGTAGCACACTACT- : 112
3’M_I612045_ : TTGACGATAATTCTTAGATGGTAGCACACTACTGTTCTCATGTATTTGTGGAGCCACACTACTA : 142

**3’S_JXLC1806-2** : ACAGCATTTAATCATTCTCCAACTATTTCATATTAGTCTTATGTATTTGTGGAGCACACTACT- : 180

3’S_Tete_virus : AACTCATTGCAATCTTATTCTTCATTATTCTTTCTGTTCTTATATAAAGTGGAGTACACTACT- : 172
3’S_Batama_virus : CTCATTACAATCTTTTTCCTCATCATTAGTTCTATTCTTATGTATCCAGTGGAGCACACTACT- : 172
3’S_I612045_ : AGCTATGCAACTCATCTTTAAATCATTTTATACTGTTCTCATGTATTTGTGGAGCACACTACT- : 179

The black padding indicates the end-conserved sequence, and the black arrow shows the mutation site in the terminal sequence of JXLC1806-2.
